# A selective and potent CXCR3 antagonist SCH 546738 attenuates the development of autoimmune diseases and delays graft rejection

**DOI:** 10.1186/10.1186/1471-2172-13-2

**Published:** 2012-01-10

**Authors:** Chung-Her Jenh, Mary Ann Cox, Long Cui, Eva-Pia Reich, Lee Sullivan, Shu-Cheng Chen, David Kinsley, Shiguang Qian, Seong Heon Kim, Stuart Rosenblum, Joseph Kozlowski, Jay S Fine, Paul J Zavodny, Daniel Lundell

**Affiliations:** 1 Department of Respiratory and Immunology, Merck Research Laboratories, 2015 Galloping Hill Road, Kenilworth, NJ 07033, USA; 2 Department of Medicinal Chemistry, Merck Research Laboratories, 2015 Galloping Hill Road, Kenilworth, NJ 07033, USA; 3 University of Pittsburgh, Starzl Transplantation Institute, Pittsburgh, PA, USA; 4 Current address: Shiguang Qian, Department of Immunology and General Surgery, Cleveland Clinic, Cleveland, OH, USA; Jay S. Fine, Boehringer Ingelheim Pharmaceuticals, Inc., Ridgefield, CT, USA

## Abstract

**Background:**

The CXCR3 receptor and its three interferon-inducible ligands (CXCL9, CXCL10 and CXCL11) have been implicated as playing a central role in directing a Th1 inflammatory response. Recent studies strongly support that the CXCR3 receptor is a very attractive therapeutic target for treating autoimmune diseases, such as rheumatoid arthritis, multiple sclerosis and psoriasis, and to prevent transplant rejection. We describe here the in vitro and in vivo pharmacological characterizations of a novel and potent small molecule CXCR3 antagonist, SCH 546738.

**Results:**

In this study, we evaluated in vitro pharmacological properties of SCH 546738 by radioligand receptor binding and human activated T cell chemotaxis assays. In vivo efficacy of SCH 546738 was determined by mouse collagen-induced arthritis, rat and mouse experimental autoimmune encephalomyelitis, and rat cardiac transplantation models. We show that SCH 546738 binds to human CXCR3 with a high affinity of 0.4 nM. In addition, SCH 546738 displaces radiolabeled CXCL10 and CXCL11 from human CXCR3 with IC_50 _ranging from 0.8 to 2.2 nM in a non-competitive manner. SCH 546738 potently and specifically inhibits CXCR3-mediated chemotaxis in human activated T cells with IC_90 _about 10 nM. SCH 546738 attenuates the disease development in mouse collagen-induced arthritis model. SCH 546738 also significantly reduces disease severity in rat and mouse experimental autoimmune encephalomyelitis models. Furthermore, SCH 546738 alone achieves dose-dependent prolongation of rat cardiac allograft survival. Most significantly, SCH 546738 in combination with CsA supports permanent engraftment.

**Conclusions:**

SCH 546738 is a novel, potent and non-competitive small molecule CXCR3 antagonist. It is efficacious in multiple preclinical disease models. These results demonstrate that therapy with CXCR3 antagonists may serve as a new strategy for treatment of autoimmune diseases, including rheumatoid arthritis and multiple sclerosis, and to prevent transplant rejection.

## Background

Leukocyte infiltration into inflammatory sites is critical for the initiation and progression of a variety of inflammatory disorders and is controlled via the activation and signaling of specific cell-surface chemoattractant receptors by their cognate protein ligands, termed chemokines. Chemokines, which are produced by a number of cell types at sites of inflammation, mediate the firm adhesion of leukocytes to activated endothelial cells, their subsequent transmigration and extravasation into the inflamed tissue, and possibly several cellular signaling pathways involved in cell activation and differentiation [1-4].

CXCR3 is a seven-transmembrane G-protein coupled chemokine receptor which has been demonstrated to play an important role in a variety of inflammatory and immunological responses. CXCR3 receptor is predominantly expressed on activated T helper 1 (Th1) cells. Its ligands, CXCL10 (IP-10), CXCL9 (MIG) and CXCL11 (I-TAC) are expressed by endothelial cells, epithelial cells and infiltrating leukocytes following stimulation by interferon (IFN)-γ or Type I IFNs and their expression is synergistically enhanced by the key pro-inflammatory mediator tumor necrosis factor (TNF)-α [5-9].

The importance of CXCR3 in leukocyte recruitment was first demonstrated in the CXCR3 knockout mouse, where the rejection of a cardiac allograft was significantly delayed compared to matched wild type animals, and where treatment of the CXCR3-deficient host with the immunosuppressive agent cyclosporine resulted in permanent allograft engraftment [10]. Transplant rejection is caused by infiltration, activation and expansion of host leukocytes in the grafted organ resulting in destruction of the donor tissue. The marked upregulation of CXCR3 ligand expression and the predominant expression of CXCR3 on infiltrating T cells during allograft rejection in human and in animal models indicate a critical role for CXCR3-dependent T cell recruitment in transplant rejection [11-13]. Similarly, the upregulation of CXCR3 ligands and the increased number of CXCR3^+ ^lymphocytes documented in chronic inflammatory diseases such as rheumatoid arthritis (RA) [14-17], multiple sclerosis (MS) [18,19] and psoriasis [20] indicates the potential importance of CXCR3-mediated leukocyte recruitment in the pathology of these conditions, and suggests the potential utility of the selective CXCR3 antagonist in the treatment and amelioration of these disorders.

To date, many different classes of small molecule CXCR3 antagonists have been discovered [21-30], and it was reported that CXCR3 antagonism reduced inflammation and cartilage damage in mouse and rat models of collagen-induced arthritis (CIA), attenuated atherosclerotic plaque formation, prolonged allograft survival, and inhibited lung metastasis [21,28,29,31-34]. In this report, we described the in vitro and in vivo pharmacological characterizations of a novel and potent CXCR3 antagonist SCH 546738 (compound 8a) [35]. So far, SCH 546738 is reported to have the highest affinity of 0.4 nM to human CXCR3 receptor. SCH 546738 inhibits CXCL10 and CXCL11 binding and human activated T cell chemotaxis with nanomolar potency. In vivo, SCH 546738 shows significant efficacy in mouse CIA and rat experimental autoimmune encephalomyelitis (EAE) model. More importantly, we show that combination of IFN-β therapy and CXCR3 inhibition has an additive effect on delaying disease onset and attenuating disease severity in the mouse EAE model. Furthermore, SCH 546738 delays graft rejection and in combination with cyclosporine, permits permanent engraftment in the rat cardiac allograft transplant model. These results demonstrate that SCH 546738 may offer a tool to evaluate the full therapeutic potential of CXCR3 antagonism in chronic inflammatory disease and preventing allograft rejection.

## Methods

### Materials

All chemokines were obtained from R & D Systems (Minneapolis, MN). ^125^I-hCXCL10 was obtained from PerkinElmer Life Science (Waltham, MA) and ^125^I-hCXCL11 from GE Healthcare Life Sciences (Piscataway, NJ). ^35^S radiolabeled SCH 535390 (a sulfonamide analog of the CXCR3 compound series) was made in the lab.

### Synthesis of SCH 546738

**Figure 1:**
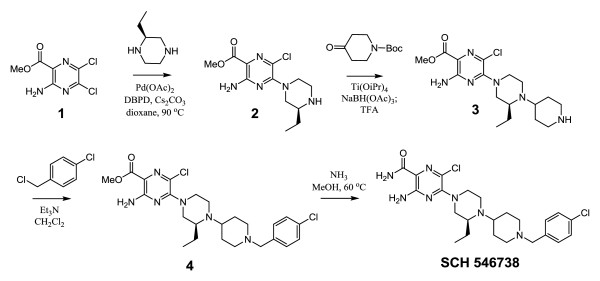
**Synthesis of SCH 546738**. Synthesis of SCH 546738 was accomplished by the method outlined with details in Methods section.

Synthesis of SCH 546738 was accomplished by the method outlined in Figure 1. The 2-chlorine of commercially available pyrazine **1** was regioselectively displaced with (*S*)(+)-2-ethylpiperazine in the presence of Pd-catalyst to afford compound **2**. Subsequent reductive amination of compound **2** with N-Boc-piperidin-4-one in the presence of Ti(O*i*Pr)_4 _followed by removal of Boc gave compound **3**. The tricyclic compound 3 was reacted with 4-chlorobenzyl chloride in the presence of excess base to provide methyl ester **4**, which was converted to SCH 546738 by heating with ammonia.

### CXCR3 expressing cells and membrane preparations

The cDNAs encoding human, mouse and rat CXCR3 were generated based on the published sequences: human (NM_001504) [5], mouse (NM_009910) [36], rat (NM_053415) [37]. The cDNAs for monkey and dog CXCR3 were cloned in the lab. All CXCR3 cDNAs were cloned into the mammalian expression vector pME18Sneo, a derivative of the SRα expression vector as described previously [38]. IL-3-dependent mouse pro-B cells Ba/F3 were transfected to express CXCR3 of different species and cell membranes were prepared as described previously [39].

### Radioligand binding assays

A scintillation proximity assay was used for radioligand competition binding assays as described previously [39] with some modifications. For each assay point, 1 µg of membrane was preincubated for 1 hr with 300 µg wheat germ agglutinin (WGA) coated SPA beads (GE Healthcare Life Sciences) in the binding buffer (50 mM HEPES, 1 mM CaCl_2_, 5 mM MgCl_2_, 125 mM NaCl, 0.002% NaN_3_, 1.0% BSA) at room temperature. The beads were spun down, resuspended in the binding buffer and transferred to a 96-well Isoplate (Wallac, Gaithersburg, MD). The indicated concentrations of ^125^I-hCXCL10, ^125^I-hCXCL11 or ^35^S-SCH 535390 with a series of titrations of SCH 546738 were added to start the reaction. After indicated reaction times at room temperature, the amount of radioactivity bound to the SPA beads was determined with a Wallac 1450 Microbeta counter (Wallac).

### Human activated T cell chemotaxis assays

The preparation of human activated T cells was performed as described previously [39]. Human peripheral blood lymphocytes were prepared by Ficoll-Hypaque centrifugation, depleted of monocytes, and stimulated for 2 days with 1 µg/ml PHA (Murex Diagnostics, Dartford, U.K.) and 100 U/ml IL-2 (Sigma, St. Louis, MO) in RPMI 1640 supplemented with 10% fetal bovine serum (FBS), 2 mM L-glutamine, 100 µg/ml streptomycin, 100 U/ml penecillin, 1% non-essential amino acids and 2 mM HEPES. Following stimulation, peripheral blood lymphocytes were cultured in above media containing 5% conditioned media (Sigma) for up to 15 days.

Human activated T cell chemotaxis assays were performed using 96-well ChemoTx^® ^microplates (Neuro Probe, Inc., Gaithersburg, MD) with a 3 µm filter as per manufacturers' instructions. Activated T cells were washed with RPMI medium twice, and then resuspended in the medium containing 20% FBS. 1.25 × 10^5 ^cells/reaction were mixed with indicated concentrations of the compound and placed on the filter. The compound and chemokines were mixed and placed in the bottom well of the ChemoTx system. After 2.5 hours incubation at 37°C/5% CO_2_, the cells were scraped off and the plate system was centrifuged for 5 minutes at 1000 RPM. The filter screen was then removed and the ChemoTx plate was inverted into a 96 well plate (Microlite + #7571 from Thermo Labsystems) with a funnel plate. The plate system was centrifuged for 5 minutes at 1000 RPM. The volume in the wells was brought to 100 μl with assay buffer and the plates were rested for approximately 15 minutes at room temperature. The number of migrated cells was measured using the Cell Titer Glo Luminescent Assay from Promega (Madison, WI) as per vendor's instructions. Chemotaxis is expressed as a chemotactic index, which is a ratio versus the one without chemokines (spontaneous migration).

### Mouse collagen-induced arthritis

Murine collagen-induced arthritis (CIA) was established as previously described [40]. Briefly, 12-week-old male B10.RIII mice (Jackson Laboratories, Bar Harbor, ME) were immunized intradermally at five sites with bovine type II collagen (Elastin Products, Owensville, MO) emulsified with an equal volume of complete Freund's adjuvant (CFA). CFA was comprised of a mixture of incomplete Freund's adjuvant (Difco, Detroit, MI) and heat-killed, freeze-dried *Mycobacteria tuberculosis *(Ministry of Agriculture, Fisheries & Food, Surrey, England). Each mouse received 300 µg/ml bovine type II collagen and 0.5 mg/ml complete Freund's adjuvant. Mice were boosted intraperitoneally with 100 µg of bovine type II collagen on day 20. Disease progression was monitored by a standardized visual scoring system with a scale from 0 to 12 reflecting the degree of swelling/redness of each paw (maximal score 3 per paw) and the number of paws (maximal 4) involved per individual animal.

### Histopathological analysis

After euthanasia, front and hind paws of the animals were dissected and fixed by immersion in 10% phosphate-buffered formalin before decalcification. Following decalcification with Cal-Rite (Richard Allen Scientific, Kalamazoo, MI), formalin fixed tissues were processed and sectioned at 5µm. Paraffin sections were stained with Hematoxylin and Eosin (H&E). The criteria of histopathological analysis was carried out as described [41]. The changes in joint structures, including cartilage destruction, bone erosion/remodelling and pannus formation were scored as follows: 0 = Normal, 1 = Minimal, 2 = Mild, 3 = Moderate, 4 = Marked, 5 = Severe. A grade of 5.5 was also added to address full-thickness cartilage breach. Cellular infiltrates and inflammation in animal joints were scored as the following: 0 = Normal, 1 = Minimal, 2 = Mild, 3 = Moderate, 4 = Marked.

### Mouse experimental autoimmune encephalomyelitis

Female C57BL/6 mice were purchased from Jackson Laboratory (Bar Harbor, ME). For immunization, 150 μg MOG35-55 peptide prepared by Princeton Biomolecules (Langhorne, PA, USA) and 300 μg killed Mycobacterium tuberculosis (Difco, Detroit, MI) were mixed in CFA (Sigma-Aldrich, St Louis, MO, USA) and injected s.c. in two 50-μl injections over the flanks on day 1. Also, 200 ng of pertussis toxin (Sigma-Aldrich, St Louis, MO) was injected i.v. on days 0 and 2. The compound was administered orally twice daily. Dosing with the compound started at day 0, 24 h prior to MOG35-55 immunization (day 1). Mice were monitored daily and assessed for clinical signs of disease in a blinded fashion according to the following criteria: 0, no signs of disease; 1, tail paralysis; 2, limp tail and hind limb weakness; 3, hind limb paralysis; 4, hind limb plus forelimb paralysis; and 5, moribund or dead. Cumulative clinical scores were calculated by adding daily scores from the day of immunization until the end of the experiment. Mean clinical scores at separate days and mean maximal scores were calculated by adding the scores of individual mice and dividing with the number of mice in each group, including mice not developing signs of EAE. All animals were used in accordance with protocols and guidelines established by institute's Animal Care and Use Committee.

### Rat experimental autoimmune encephalomyelitis

Male Lewis rats challenged by injection of 50 µl (30 mg) of a guinea pig spinal cord homogenate in CFA into one footpad. The animals were treated starting at day 0 and oral dosing continued throughout the 3-week disease course, with varying amounts of SCH 546738 in 0.4% methylcellulose (MC) p.o. Animals were scored for disease severity: 0, no clinical signs; 1, flaccid tail; 2, hind limb weakness; 3, complete hind limb paralysis; 4, complete hind limb paralysis, forelimb weakness or paralysis; 5, death.

### Statistical analysis

For CIA and EAE models, unpaired t-tests were performed using GraphPad InStat version 5.0.1 for Windows 98, GraphPad Software, San Diego California USA (http://www.graphpad.com). Statistical significance was evaluated by comparing the vehicle-treated group with the experimental group using unpaired t-test. Differences were considered significant when p values were <0.05.

### Cardiac transplantation in rats

Cardiac graft of ACI (RT1a) rats was heterotopically transplanted into the abdominal cavity of Lewis (RT1^l^) recipients employing a microvascular surgical technique as described [42]. The grafts were monitored daily by abdominal palpation, and the complete cessation of heart contraction was defined as graft rejection. SCH 546738 or 0.4% methylcellulose (vehicle) was orally administered at the indicated dose (0.2 ml) twice a day, starting on the day before transplantation until the day of graft rejection. To test whether SCH 546738 enhanced the effect of conventional immunosuppressive reagent, the recipients were received treatment with subtherapeutic dose of CsA for one week combined with treatment with SCH 546738. Graft survival was analyzed using the log-rank test. The parametric data were analyzed by Student t test (2-tailed) using GraphPad InStat version 5.0.1 for Windows 98, GraphPad Software, San Diego California USA (http://www.graphpad.com). p < 0.05 was considered statistically significant.

## Results

To identify CXCR3 antagonists, we have generated a mouse Pro-B cell line Ba/F3 stably expressing a high level of human CXCR3 receptor. The membranes were prepared for establishing a sensitive binding assay using [^125^I]hCXCL10 based on the scintillation proximity assay [39]. From high throughput screening of small molecule compound libraries, several lead compounds were discovered [43]. Through the optimization of the lead compound, we have found SCH 546738 (compound 8a) [35] to be a selective and potent CXCR3 antagonist with a good PK for in vivo studies. Its structure is shown in Figure 1.

### Affinity of SCH 546738 for CXCR3 receptor

The affinity of SCH 546738 binding to human CXCR3 receptor was determined by competition binding analysis using ^35^S radiolabeled SCH 535390 (a sulfonamide analog of the CXCR3 compound series with a K_d _of 0.6 nM) as a competitive tracer. In multiple experiments, the affinity constant (K_i_) of SCH 546738 binding to human CXCR3 receptor was determined to be 0.4 nM (data not shown).

### Inhibition of CXCL10 and CXCL11 binding to CXCR3 receptor

**Figure 2:**
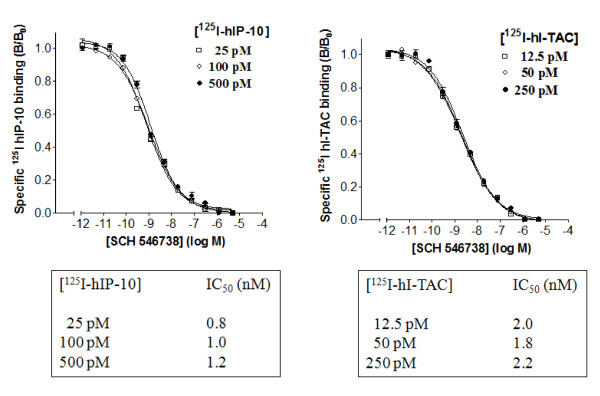
**Effect of concentrations of [^125^I]hCXCL10 and [^125^I]hCXCL11 on the IC_50 _of SCH 546738 binding to human CXCR3 receptor**. The ability of SCH 546738 to compete the binding of [^125^I]hCXCL10 and [^125^I]hCXCL11 to human CXCR3 receptor was determined using various concentrations of [^125^I]hCXCL10 and [^125^I]hCXCL11 as described in Methods. After 3 hr reaction, specific counts relative to input counts (B/B_0_) in the presence of increasing concentrations of SCH 546738 are plotted. The IC_50 _for each concentration of [^125^I]hCXCL10 and [^125^I]hCXCL11 are shown.

Competition of human CXCL10 and CXCL11 binding to human CXCR3 by SCH 546738 was determined at various concentrations of [^125^I]hCXCL10 and [^125^I]hCXCL11 around the K_d _(50-100 pM) for the receptor. The IC_50 _of SCH 546738 is constant (~1 or 2 nM) and independent of the input concentrations of either [^125^I]hCXCL10 (25-500 pM) or [^125^I]hCXCL11 (12.5-250 pM) (Figure 2), respectively. These results indicate that SCH 546738 is a non-competitive antagonist of both CXCL10 and CXCL11 binding to CXCR3, suggesting that SCH 546738 binds to CXCR3 receptor at an allosteric site and change its conformation which prevents the binding of both CXCL10 and CXCL11.

It is important to investigate species specificity of SCH 546738 to design in vivo preclinical studies. As shown in Table 1, SCH 546738 has strong cross-species activities with IC_50 _of 1.3 nM, 6.4 nM, 5.9 nM and 4.2 nM in inhibiting the binding of [^125^I]hCXCL10 to CXCR3 of monkey, dog, mouse and rat origin, respectively.

**Table 1:** Effect of SCH 546738 on CXCR3 from various species

Species	Human	Monkey	Dog	Mouse	Rat
IC50 (nM) (CXCL10)	0.8	1.3	6.4	5.9	4.2

Competition binding assays using [^125^I]hCXCL10 were carried out as described in Methods with membranes prepared from Ba/F3 cells expressing human, monkey, dog, mouse and rat CXCR3 receptors.

### Functional inhibition of CXCR3-mediated chemotaxis

**Figure 3:**
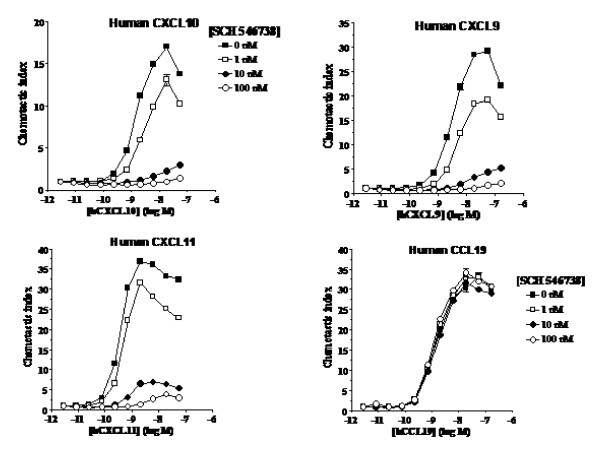
**Effect of SCH 546738 on human Activated T cell chemotaxis induced by CXCL10, CXCL9, CXCL11 and CCL19**. Human peripheral blood lymphocytes were prepared by Ficoll-Hypaque centrifugation, depleted of monocytes, and stimulated by PHA/IL-2. After 7-9 days of stimulation, activated T cell chemotaxis assay was carried out as described in Methods. The medium containing 20% fetal bovine serum was used for all dilutions of chemokines and compounds. Various concentrations of each indicated chemokine were added to the bottom wells in the presence of fixed concentrations of SCH 546738 (1, 10, 100 nM) (added both on the filter with cells and in the bottom wells). Chemotaxis of each sample is expressed relative to spontaneous response (without chemokines) as chemotactic index.

The functional activity of SCH 546738 was investigated by CXCR3-mediated chemotaxis assays using human activated T cells. SCH 546738 at fixed concentrations of 1, 10 or 100 nM was evaluated for its ability to inhibit human activated T cell chemotaxis induced by various concentrations of the three CXCR3 ligands CXCL9, CXCL10 and CXCL11 and the CCR7 ligand CCL19 (MIP-3β). SCH 546738 at 10 nM inhibited T cell chemotaxis induced by all three CXCR3 ligands about 90% (Figure 3). In contrast, SCH 546738 did not affect T cell chemotaxis induced by the CCR7 ligand CCL19. Furthermore, SCH 546738 inhibited T cell chemotaxis induced by the three CXCR3 ligand among all tested ligand concentrations in an insurmountable manner, suggesting that SCH 546738 is a non-competitive antagonist, as has been characterized in the competition binding analyses (Figure 2). It is critical to have a nonocompetitive antagonist which will inhibit binding of multiple endogenous ligands and inhibit its function (or activation) at any possible high local concentration of the ligand in the disease stage.

### Biochemical selectivity and pharmacokinetic properties

**Figure 4:**
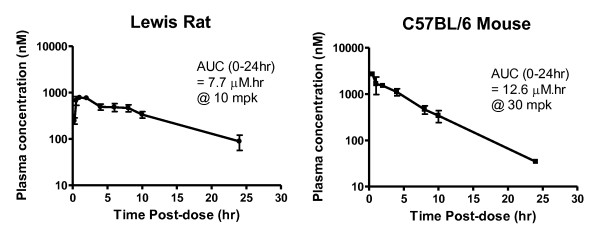
**Plasma concentration versus time profiles of SCH 546738 in Lewis rat and C57BL/6 mouse**. SCH 546738 in 0.4% methylcellulose was administered orally at 10 mg/kg (mpk) in Lewis rats or 30 mpk in C57BL/6 mice. The plasma concentration of SCH 546738 in the blood was calculated as the mean of 3 animals (n = 3) at indicated time points post-dose. Their AUC from 0 to 24 hr is also calculated and indicated.

SCH 546738 was tested at concentrations of 1-10 μM against a panel of 49 GPCR binding assays. Most of the assays were not affected by SCH 546738 (Table 2). These results indicate that SCH 546738 is a highly selective antagonist of CXCR3. In addition, SCH 546738 has a favourable pharmacokinetic profile in rodents. Figure 4 shows the plasma concentrations of SCH 546738 in Lewis rat and C57BL/6 mouse over 24 hr post-dose. The AUC (0-24 hr) is 7.7 μM.hr in Lewis rat @ 10 mg/kg (mpk) and is 12.6 μM.hr in C57BL/6 mouse @ 30 mpk. Therefore, SCH 546738 is suitable for in vivo preclinical studies.

**Table 2:** GPCR counterscreens of SCH 546738

**Assay **	**Inhibition (%) **	**Assay **	**Inhibition (%) **
Adenosine A_1_	0% @ 4 μM	Muscarinic M_1_	22% @ 4 μM
Adenosine A_2A_	3% @ 4 μM	Muscarinic M_2_	52% @ 4 μM
Adrenergic α_1A_	11% @ 2 μM	Muscarinic M_3_	27% @ 4 μM
Adrenergic α_1B_	25% @ 2 μM	Muscarinic M_4_	30% @ 4 μM
Adrenergic α_2A_	45% @ 1 μM	Muscarinic M_5_	28% @ 4 μM
Adrenergic α_2B_	-2% @ 2 μM	Neurokinin NK_1_	15% @ 2 μM
Adrenergic α_2C_	76% @ 1 μM	Neurokinin NK_2_	0% @ 2 μM
CCR1	9% @ 10 μM	Neurokinin NK_3_	0% @ 2 μM
CCR2	9% @ 10 μM	Neuropeptide Y (Y_1_)	4% @ 4 μM
CCR3	17% @ 10 μM	Neuropeptide Y (Y_2_)	-9% @ 4 μM
CCR5	10% @ 10 μM	Neuropeptide Y (Y_4_)	-2% @ 4 μM
CCR7	0% @ 10 μM	Neuropeptide Y (Y_5_)	12% @ 4 μM
CXCR1	13% @ 10 μM	Nociceptin NOP_1_	-21% @ 4 μM
CXCR2	20% @ 10 μM	Delta opiate	12% @ 4 μM
Dopamine D_1_	6% @ 4 μM	Kappa opiate	7% @ 4 μM
Dopamine D_2_	41% @ 4 μM	Mu opiate	22% @ 4 μM
Dopamine D_3_	18% @ 10 μM	Prokineticin PKR_1_	-1% @ 4 μM
Dopamine D_4.2_	18% @ 10 μM	Prokineticin PKR_2_	-5% @ 4 μM
Histamine H_1_	46% @ 2 μM	Purinergic P2Y_1_	16% @ 4 μM
Histamine H_3_	29% @ 2 μM	Purinergic P2Y1_2_	10% @ 4 μM
MCHR_1_	0% @ 10 μM	Serotonergic 5HT_1A_	20% @ 4 μM
MRGX_1_	1% @ 4 μM	Vasopressin (V_1a_)	7% @ 4 μM
MRGX_2_	25% @ 4 μM	Vasopressin (V_1b_)	15% @ 4 μM
		Vasopressin (V_2_)	18% @ 4 μM
		Vasopressin (oxytocin)	12% @ 4 μM
		VR1N	0% @ 4 μM

SCH 546738 was tested at the indicated concentrations in a variety of GPCR assays according to established protocols.

### Administration of SCH 546738 attenuates disease in mouse collagen-induced arthritis and protects joint structure

**Figure 5:**
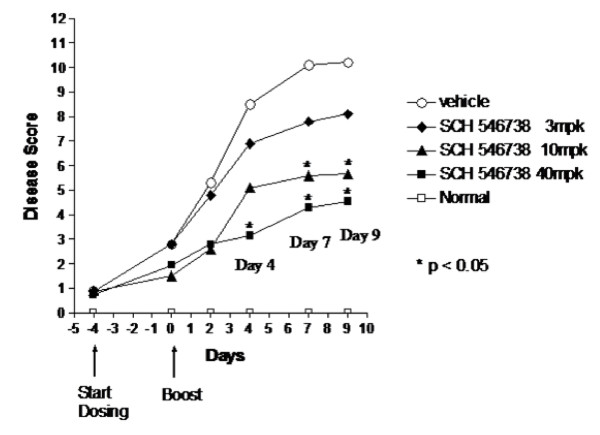
**SCH 546738 attenuates disease in mouse collagen-induced arthritis**. SCH 546738 in 0.4% methylcellulose was administered orally twice daily at 3, 10 and 40 mg/kg (mpk). Dosing was initiated 16 days (day -4) postimmunization and continued through day 9. The disease score was significantly decreased in SCH 546738-treated animals as compared with vehicle-treated animals (* p < 0.05, two-tailed t test) at day 4, 7 and 9.

Collagen-induced arthritis (CIA) was induced in male B10.RIII mice by immunization with bovine collagen type II (BC II) which resulted in the development of poly-arthritis in the paw. Sixteen days later which was 4 days prior to receiving a BC II boost (day -4); mice were randomized into treatment groups with approximately 10% of the animals in each group having developed at least one swollen paw. Oral twice daily treatment with SCH 546738 was initiated at this time (day -4) and continued through day 9, with a BC II antigen boost on day 0. Figure 5 shows that SCH 546738 attenuated disease development in a dose-dependent fashion, with significant reduction of the disease score evident at 40 mpk on days 4, 7 and 9, while it protected significantly on days 7 and 9 at 10 mpk. SCH 546738 administration at 3 mpk had no statistically significant effect on disease severity.

**Figure 6:**
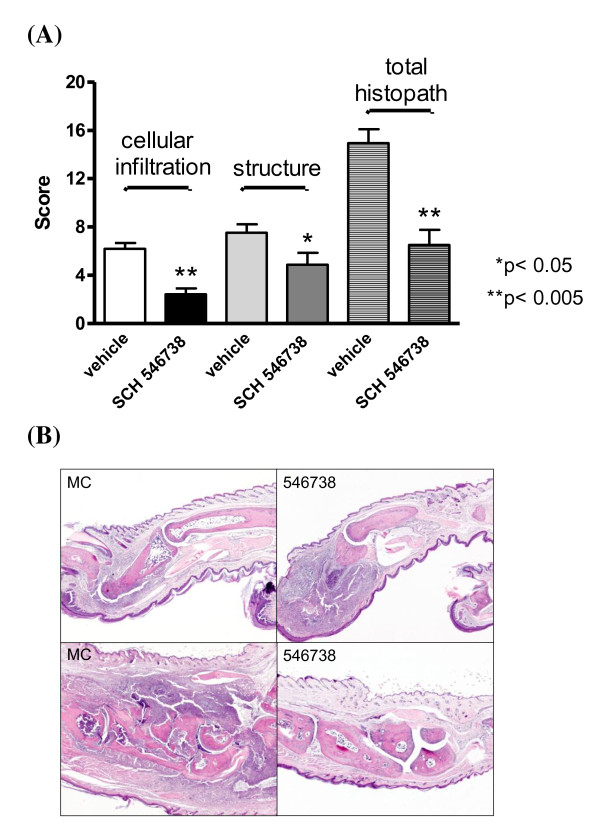
**SCH 546738 in mCIA: Histopathological analysis of paws on day 9**. Paws collected at day 9 from the vehicle (0.4% methylcellulose) and 40 mpk SCH 546738 groups of two independent experiments were analyzed by histopathology. (A) compares the histopathological scores of both groups of tissues. Both leukocyte infiltration into the joint and the structural damage to the bone and cartilage was significantly attenuated in SCH 546738-treated animals (* p < 0.05; ** p < 0.005, two-tailed t test). Example images of paw tissue sections collected from both groups of animals are shown in (B) (top, phalanges area; bottom, tarsal area). Massive cellular infiltrates and bone/cartilage erosions were evident in both tarsal and phalanges areas of the vehicle treated mouse paw (left panels). In contrast, cellular infiltrates were mainly observed in the phalanges region (right top), and rarely in the tarsal region (right bottom) of SCH546738-treated animals.

Paws collected on day 9 from the vehicle and 40 mpk SCH 546738 groups of two independent experiments were analyzed by histopathology. Statistical analysis of the combined histopathology scores demonstrated that in animals treated with 40 mpk SCH 546738, both leukocyte infiltration into the joint and the structural damage to the bone and cartilage was significantly attenuated (Figure 6). This data demonstrates that therapeutic treatment with a CXCR3 antagonist significantly impairs the development of disease in an animal model of rheumatoid arthritis, and supports the clinical development of SCH 546738 in this disease.

### Administration of SCH 546738 reduces disease in experimental autoimmune encephalomyelitis

**Figure 7:**
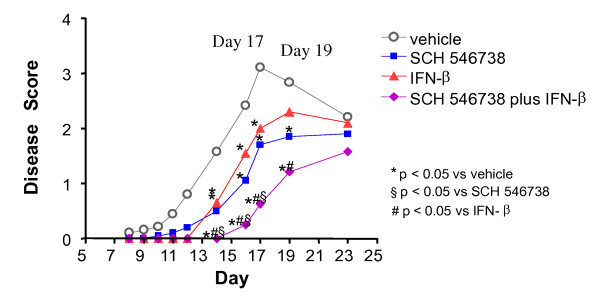
**Combination of IFN-β therapy and CXCR3 inhibition has an additive effect on delaying disease onset and attenuating disease severity in the mouse EAE model**. IFN-β was administered at 1700 ng by daily intramuscular injection and SCH 546738 was orally twice daily at 30 mpk. The mouse EAE was conducted as described in Methods. Treatment with either IFN-β or SCH 546738 alone or the combination significantly delayed disease onset and attenuated disease severity (p < 0.05, two-tailed t test) at day 16, 17 and 19.

Experimental autoimmune encephalomyelitis (EAE) is an animal model for human MS and development of disease is dependent on T cell infiltration into the CNS. In the murine model of EAE, SCH 546738 was tested in combination with interferon-β (IFN-β), a current first-line therapeutic for the amelioration of relapsing-remitting MS. C57BL/6 mice were primed by intravenous injection of pertussis toxin on day 0 and day 2. EAE was induced on day 1 by subcutaneous injection of the myelin peptide MOG 35-55 emulsified in CFA in the back of primed mice. Disease progression was monitored by a scoring system as described in Methods. IFN-β administered at 1700 ng by daily intramuscular injection significantly delayed disease onset and attenuated disease severity at peak of disease compared to vehicle treated animals (Figure 7). Similarly, SCH 546738 at 30 mpk orally twice daily delayed disease onset and attenuated disease severity on days 17 and 19 (Figure 7). Combination treatment with SCH 546738 and IFN-β had a significant additive effect in delaying disease onset and attenuating disease severity compared to treatment with either SCH 546738 or IFN-β alone (Figure 7) suggesting that a CXCR3 antagonist may offer substantial 'add-on' efficacy onto existing IFN-β therapy and further delay the occurrence of relapses in MS patients. In addition, EAE was induced in Lewis rats by subcutaneous injections of guinea pig spinal cord emulsified in CFA into one hind paw. SCH 546738 reduced the severity of the disease in a dose-dependent manner as well (data not shown).

### Inhibition of CXCR3 delays graft rejection and in combination with cyclosporine, permits permanent engraftment

**Figure 8:**
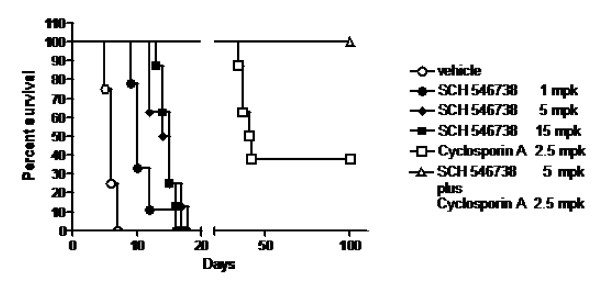
**SCH 546738 delays graft rejection and in combination with cyclosporine, permits permanent engraftment in the rat cardiac allograft transplant model**. SCH 546738 was administered orally twice daily at 1, 5 and 15 mpk. Cyclosporine was administered daily at 2.5 mpk. In the combination study, 5 mpk SCH 546738 and 2.5 mpk cyclosporine were administered. SCH 546738 significantly increased the mean survival time of the graft at 1 mpk (MST = 11 days; p < 0.05), 5 mpk (MST = 14 days; p < 0.05) and 15 mpk (MST = 14.9 days; p < 0.05) when compared with the vehicle control (MST = 6 days). Graft survival was analyzed using the log-rank test.

Published data demonstrated that in the CXCR3 knockout mouse rejection of cardiac allografts was significantly delayed [10]. Based on this observation SCH 546738 was tested at various doses by twice daily oral administration in a rat cardiac allograft model starting at the day of transplantation. SCH 546738 significantly increased the mean survival time of the graft at 1 mpk (MST = 11 days) when compared to the vehicle control (MST = 6 days), and further delayed graft rejection at a dose of 5 mpk (MST = 14 days) (Figure 8). Cyclosporine is the current gold standard in organ transplant therapies in human. A cyclosporine dose response was conducted earlier in a rat cardiac allograft model and 2.5 mpk of cyclosporine is a low and suboptimal dose (data not shown). Figure 8 shows that cyclosporine significantly delayed graft rejection in the rat model at a daily suboptimal dose of 2.5 mpk and permitted the permanent engraftment of approximately 40% of the grafts (>100 days graft survival). In combination with 2.5 mpk cyclosporine a suboptimal dose of 5 mpk SCH 546738 twice daily increased the rate of permanent engraftment to 100% (Figure 8). These data indicate that the selective inhibition of CXCR3 would have a beneficial effect on allograft survival and may offer the possibility of reducing the dose of cyclosporine used in patients, thereby limiting the potential for serious side effects.

## Discussion

The CXCR3 receptor and its three interferon-inducible ligands (CXCL9, CXCL10 and CXCL11) have been implicated in several Th1-mediated inflammatory diseases. Recently, the efficacy of the anti-IP-10 antibody MDX-1100 reported in a phase 2 clinical trial for RA [44] reinforced the crucial role of the CXCL10-CXCR3 axis in this disease, and the therapeutic potential of small molecule CXCR3 antagonists [45]. So far, only one of the CXCR3 antagonists, AMG487 (T487), progressed to Phase II clinical trials but has been halted because of lack of efficacy. Since this may have been due to variability in drug exposure, it is clear that this failure is not a misrepresentation of CXCR3 as a drug target. In this regard, SCH 546738 is a small molecule non-competitive CXCR3 antagonist with much higher affinity than AMG487 and therefore may have better chance to achieve the in vivo efficacy.

In the mouse CIA model, SCH 546738 is efficacious in reducing disease development by attenuating leukocyte infiltration into the joint and the structural damage to the bone and cartilage. It is of interest to note that SCH 546738 demonstrated efficacy even though dosing was started after the disease process was initiated and when mice had already started to show signs of paw swelling. It was reported that T487 reduced inflammation and cartilage damage in mouse and rat models of CIA [21]. In rat adjuvant arthritis, blockade of CXCR3 by anti-CXCR3 mAb significantly inhibits T cell infiltration of arthritic joints and reduces the severity of arthritis [46]. All these data directly demonstrate an important role of CXCR3 in the development of arthritis and CXCR3 blockade reduces the disease severity in the arthritis. It is likely that small molecule CXCR3 antagonists may achieve the efficacy of the anti-IP-10 antibody MDX-1100 reported in a phase 2 clinical trial for RA.

The available functional data for the role of CXCR3 and its ligands in EAE are contradictory. Different investigators have reported conflicting results when using IP-10^-/- ^mice, anti-IP-10 antibody, anti-sense RNA and vaccines [47,48]. The recent results from CXCR3^-/- ^mice show that CXCR3 is not required for the recruitment of immune cells to the CNS in MOG-EAE. The work by Liu et al. [49] showed exacerbation of EAE disease in CXCR3^-/- ^mice and with neutralizing anti-CXCR3 Abs. It indicates that the exacerbation in the CXCR3^-/- ^mice correlates with enhanced effector T cell proliferation and reduced peripheral and CNS expression of IFN-γ, but with no impact on leukocyte migration to CNS. A subsequent study by Muller et al. [50] showed that CXCR3^-/- ^mice had more severe chronic disease with increased demyelination and axonal damage, although the number of CD4+ and CD8+ T cells infiltrating the CNS were similar in CXCR3^-/- ^and wild type mice. In contrast to MOG-EAE, CXCR3 appears to promote the lymphocyte accumulation inside the CNS in some virus-induced demyelinating disease models [51]. This may point to disease-specific functions of CXCR3 and its ligands, which can vary depending on the nature of the pathogenic insult. These varied results probably reflect the complex and perhaps divergent roles for the chemokine system in the pathogenesis of EAE and virus-induced neuroinflammatory diseases. Recently, a nonspecific small molecule antagonist of CCR5, CCR2 and CXCR3 (TAK-779) was reported to reduce incidence and severity of EAE by decreasing migration of inflammatory cells into the CNS [52]. Our study is the first report that a specific small molecule CXCR3 antagonist SCH 546738 consistently inhibits both mouse and rat EAE clinical disease with no evidence of exacerbation. Furthermore, combination of IFN-β therapy and CXCR3 inhibition has an additive effect on delaying disease onset and attenuating disease severity in the mouse EAE model. At least for small molecule antagonists including SCH 546738, the beneficial effect of CXCR3 blockade has been observed in EAE. Maybe studies using CXCR3^-/- ^mice and neutralizing anti-CXCR3 Abs offer some hints as to other possible function of CXCR3 receptor and its ligands. Beyond leukocyte recruitment, CXCR3 may modulate T cell IFN-γ production, regulation between Th1 vs. Th17 cells, or control T cells at the perivascular space in the CNS. It is not unlikely that a small molecule antagonist, a neutralizing antibody or a genetic deletion can perturb a receptor's activity in different ways, leading to different conclusion about the protein's biological function.

The role of CXCR3 in leukocyte recruitment was first demonstrated in the CXCR3 knockout mouse in year 2000, where the rejection of a cardiac allograft was significantly delayed, and resulted in permanent allograft engraftment with cyclosporine [10]. In addition, lack of CXCL10 in the graft led to prolonged allograft survival [53]. However, two recent studies published in 2008 [54,55] questioned the importance of CXCR3 in allograft rejection and found moderate to little increase in graft survival using CXCR3^-/- ^mice or small molecule CXCR3 antagonist MRL-957 and anti-CXCR3 antibody targeting in human CXCR3 knock-in mice. These two studies conclude that CXCR3 is not essential for leukocyte recruitment in the cardiac allograft rejection. In contrast, Uppaluri et al. [56] demonstrates that a CXCR3 blocking antibody significantly prolonged both cardiac and islet allograft survival, and induced long-term graft survival greater than 100 days when combined with rapamycin. In 2009, one study shows that TAK-779 attenuates cardiac allograft vasculopathy in part by reducing CCR5^+ ^and CXCR3^+ ^T lymphocyte subset infiltration into the graft [57]. The other study by Rosenblum et al. [58] shows that small molecule CXCR3 antagonist AMG1237845 prolongs allograft survival; however, it does not inhibit leukocyte recruitment into the graft. The difference in the contribution of CXCR3 to mouse allograft rejection observed in similar models in different laboratories can not be explained by current data sets and additional experiments are required to clarify these conflicting results.

In the rat cardiac allograft transplant model, a small molecule CXCR3 antagonist TLRK-A was reported to prolong graft survival, but was active only in combination with cyclosporine [59]. However, another small molecule CXCR3 antagonist NIBR2130 did not prolong graft survival [55]. In this study, we demonstrate that SCH 546738 delays graft rejection and in combination with cyclosporine, permits permanent engraftment in the rat cardiac allograft transplant model.

In summary, our study demonstrates that administration of SCH 546738 attenuates disease in mouse CIA, rat and mouse EAE, and rat cardiac allograft rejection. Combination of IFN-β therapy and SCH 546738 has an additive effect in the mouse EAE model. Furthermore, in combination with cyclosporine, SCH 546738 permits permanent engraftment in the rat cardiac allograft transplant model.

The findings from our study and others indicate that targeting the CXCR3 receptor by small molecule antagonists and antibodies can be a promising approach to RA. Since the results from CXCR3 inhibition in EAE and allograft rejection remains contradictory, we need to better understand the roles of the chemokine system operating in the pathogenesis of EAE and allograft rejection that truly reflects the molecular mechanism in human diseases and enhance the chance of success in human clinical trials.

## Conclusions

In the present study, we describe the in vitro and in vivo pharmacological characterizations of a novel and potent small molecule CXCR3 antagonist, SCH 546738. It binds to human CXCR3 with an affinity of 0.4 nM, which is the most potent small molecule CXCR3 antagonist reported so far. Competition binding studies show that SCH 546738 is able to displace radiolabeled CXCL10 and CXCL11 from human CXCR3 with high affinity (IC_50 _ranged from 0.8 to 2.2 nM) in a non-competitive manner. In addition, SCH 546738 has strong cross-species activity with IC_50 _of 1.3 nM, 5.9 nM, 4.2 nM and 6.4 nM for monkey, mouse, rat and dog CXCR3 receptor, respectively. SCH 546738 potently and specifically inhibits CXCR3-mediated chemotaxis in human activated T cells with IC_90 _about 10 nM. SCH 546738 has a favorable pharmacokinetic profile in rodents. We utilized multiple preclinical disease models relevant to human rheumatoid arthritis, multiple sclerosis, transplantation to assess in vivo efficacy of SCH 546738. We demonstrate that SCH 546738 attenuates the disease development in mouse collagen-induced arthritis model by decreasing both leukocyte infiltration into the joint and the structural damage to the bone and cartilage. SCH 546738 also significantly reduces disease severity in rat experimental autoimmune encephalomyelitis model, and in combination with IFN-β in mouse experimental autoimmune encephalomyelitis model. Furthermore, SCH 546738 alone achieves dose-dependent prolongation of rat cardiac allograft survival. Most significantly, SCH 546738 in combination with cyclosporine supports permanent engraftment. Taken together, the results show that therapy with potent small molecule CXCR3 antagonists may serve as a new strategy for treatment of autoimmune diseases, including rheumatoid arthritis and multiple sclerosis, and to prevent transplant rejection.

## Abbreviations

IP-10: IFN-γ inducible protein 10; MIG: monokine induced by IFN-γ; I-TAC: interferon-inducible T cell alpha chemoattractant; RA: rheumatoid arthritis; MS: multiple sclerosis; CIA: collagen-induced arthritis; EAE: experimental autoimmune encephalomyelitis; MOG: myelin oligodendrocyte glycoprotein; MIP-3β: macrophage inflammatory protein-3β; GPCR: G protein-coupled receptor.

## Competing interests

All the authors (except SQ) are employees of Merck and the work was funded by Merck.

## Authors' contributions

CHJ performed radioligand binding assays, coordinated the study and wrote the manuscript. MAC performed human activated T cell chemotaxis assay, cloned monkey and dog CXCR3 cDNAs and made membrane preparations. LC and EPR carried out mouse and rat experimental autoimmune encephalomyelitis models. LS carried out mouse collagen-induced arthritis model. SCC and DK performed histopathological analysis of samples from mouse collagen-induced arthritis model. SQ carried out cardiac transplantation in rats. SHK, SR and JK discovered and synthesized SCH 546738. JSF, PJZ and DL supervised animal models, participated in the design of the study and supported the preparation of the manuscript. All authors read and approved the final manuscript.
